# Battle Against Cancer: An Everlasting Saga of p53

**DOI:** 10.3390/ijms151222109

**Published:** 2014-12-01

**Authors:** Qian Hao, William C. Cho

**Affiliations:** 1 School of Continuing Studies, Tulane University, New Orleans, LA 70118, USA; 2 Department of Clinical Oncology, Queen Elizabeth Hospital, Hong Kong 999077, China

**Keywords:** p53, mutant p53, MDM2, ribosomal stress, transcription, cancer chemotherapeutics

## Abstract

Cancer is one of the most life-threatening diseases characterized by uncontrolled growth and spread of malignant cells. The tumor suppressor p53 is the master regulator of tumor cell growth and proliferation. In response to various stress signals, p53 can be activated and transcriptionally induces a myriad of target genes, including both protein-encoding and non-coding genes, controlling cell cycle progression, DNA repair, senescence, apoptosis, autophagy and metabolism of tumor cells. However, around 50% of human cancers harbor mutant p53 and, in the majority of the remaining cancers, p53 is inactivated through multiple mechanisms. Herein, we review the recent progress in understanding the molecular basis of p53 signaling, particularly the newly identified ribosomal stress—p53 pathway, and the development of chemotherapeutics via activating wild-type p53 or restoring mutant p53 functions in cancer. A full understanding of p53 regulation will aid the development of effective cancer treatments.

## 1. Introduction

**Figure 1:**
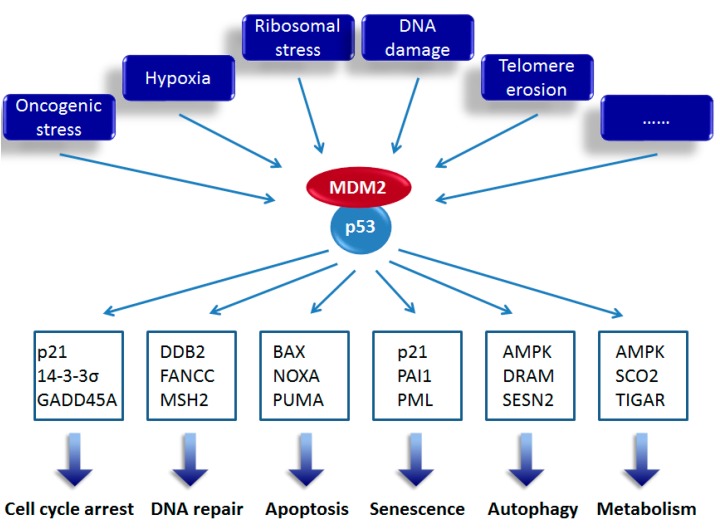
Summary of the p53 pathway. Under normal conditions, the major negative regulator, MDM2, binds to p53 and inhibits its transcriptional activity. In response to various stress signals, including oncogenic stress, hypoxia, ribosomal stress, DNA damage, telomere erosion and others, p53 can be activated, principally through abrogating MDM2 inhibition, and transcriptionally induces the expression of an array of target genes leading to different cellular outcomes. Three representative target genes dictating each cell fate are listed.

Tumorigenesis is promoted by deregulation or mutation of genes involved in cell division control. The tumor suppressor p53 is the most prominent and extensively studied example due to its dominating effect on tumor cell growth and proliferation [1,2,3,4,5]. Since its discovery in 1979, *p53* has been regarded as an oncogene as its overexpression could immortalize primary cells, and could promote transformation of such cells through cooperating with other established oncoproteins, such as H-RAS, a GTPase involved in many signal transduction pathways [6,7,8]. Although there was discussion during the 1980s that *p53* might possess tumor suppressive activity, *p53* was not redefined as a tumor suppressor gene until the wild-type *p53* sequence was confirmed at the end of the 1980s [9,10]. Baker *et al.* [11] found that wild-type *p53* is frequently lost or mutated in human colorectal cancer, and that these tumor cells therefore no longer retain wild-type p53. This could be the reason why the mutant, but not wild-type, *p53* was firstly cloned from cancer cells. To date, tens of thousands of studies have demonstrated that p53 expands its tumor suppressive functions to most, if not all, aspects of cancer development. p53 is a transcriptional factor that regulates a large number of target genes involved in cell cycle arrest, apoptosis, senescence, autophagy and metabolism [12] (Figure 1), although some effects of p53 are independent of its transcriptional activity [13,14,15]. Thus, tumor cells need to employ additional mechanisms to negate p53 functions in favor of their survival. Indeed, more than 50% of human cancers harbor mutant p53 [16,17,18], while in most of the remaining cancers, p53 activity is markedly impaired through, for instance, overexpression of the E3-ligase MDM2 (murine double minute 2, also known as HDM2 for its human ortholog) [19,20]. MDM2 is a major inhibitor of p53 found to be overexpressed or amplified in multiple cancers and physically interact with both *N*- and *C*-termini of p53 [21]. At least three principal mechanisms have been demonstrated for MDM2-mediated inactivation of p53; MDM2: abrogates transcriptional activity of p53 through directly binding to its *N*-terminal transactivation domain [22]; destabilizes p53 by promoting its poly-ubiquitination and proteasomal degradation [23,24,25]; and prompts nuclear export of p53 by inducing its mono-ubiquitination [26]. The importance of MDM2-mediated p53 inactivation has been verified by elegant studies showing that genetic disruption of the *p53* gene rescues the lethal phenotype of *Mdm2* knock-out mice [27,28] and that inhibition of MDM2 results in robust p53 activation in tumor cells [29,30]. Importantly, several other mechanisms have been demonstrated to overcome the oncogenic effect of p53 inactivation. For instance, depletion of Skp2, an E3-ligase of the G1 Cyclin-Cdk protein kinase inhibitor p27, has been found to prevent prostate tumorigenesis caused by inactivation of p53 and another tumor suppressor pRb [31].

## 2. Activation of p53 upon Ribosomal Stress

As mentioned above, the tumor suppressor p53 is often inactivated in tumor cells, thus, reactivation of p53 upon stress signaling is an important strategy to inhibit tumor cell growth and proliferation. For example, DNA damage triggers p53 phosphorylation by ATM, ATR, DNA-PK, Chk, Chk2, *etc.* [32,33,34] and acetylation by p300/CBP, PCAF, hMOF and Tip60 [35,36,37,38,39], which disrupts the interaction of MDM2 and p53, consequently leading to p53 activation [40]. In addition, p53 can also be stabilized and activated in response to hypoxia or low oxygen tension. HIF1α is the first candidate that was found to directly associate with and stabilize p53 under hypoxia [41,42]. Other studies have also shown that hypoxia stimulates ATR-CHK1 cascade-mediated phosphorylation of p53 [43] and the MDM2 partner, MDMX [44], resulting in p53 activation.

Over the past decade, ribosomal stress (also known as nucleolar stress) has emerged and been gradually appreciated as an essential cellular pressure that can induce p53 activation and resultant tumor growth inhibition [30,45]. Ribosome biogenesis, the process of producing the cellular translational machinery—the ribosome—requires a coordinated network of ribosomal proteins (RPs), rRNAs and non-ribosomal factors. Principally, RPs are synthesized in the cytoplasm and transported to the nucleolus; rRNAs are synthesized in the nucleolus or nucleoplasm (only for 5S rRNA). The pre-ribosomes are assembled in the nucleolus with the assistance of non-ribosomal factors, and consequently exported to the cytoplasm forming the mature ribosome. Stimuli that perturb any step of this process can cause ribosomal stress [30,45]. These stimuli are categorized into three groups: (1) chemical agents inhibiting rRNA synthesis; (2) nutrient depletion limiting energy availability for ribosome biogenesis; and (3) dysfunction of RP-encoding genes or non-ribosomal genes required for rRNA synthesis or ribosome assembly [45]. One may raise the question of how ribosomal stress can trigger p53 activation. A series of studies have demonstrated that ribosomal stress results in the accumulation of ribosome-free RPs in the nucleoplasm, and as such, they bind to MDM2 and inhibit its E3-ligase activity towards p53 [30,45].

Although the association of RPL5 and MDM2 was initially identified in 1994 [46], the physiological function of this interaction had been unclear until three independent groups found that several RPs, including RPL11, RPL5 and RPL23, could directly bind to MDM2 and inhibit MDM2-mediated p53 ubiquitination and proteasomal degradation in response to ribosomal stress triggered by actinomycin D (Act D) [47,48,49,50,51]. Thereafter, increasing attention has been drawn to the newly discovered field and a dozen of RPs, including RPL6 [52], RPL26 [53], RPL37 [54], RPS3 [55], RPS7 [56,57], RPS14 [58], RPS15 [54], RPS20 [54], RPS25 [59], RPS26 [60], RPS27 [61] and RPS27A [62], have been identified to interact with MDM2 and repress its inhibitory function towards p53 during the past ten years. Although most of these RPs can stabilize p53 by preventing its ubiquitination and degradation from MDM2, some of them have been found to activate p53 through additional mechanisms. For instance, RPL26 can bind to the 5' untranslated region (UTR) of p53 mRNA and augment p53 translation [63]. The association of MDM2 with RPL26 was shown to attenuate the binding of RPL26 to p53 mRNA, as thus suppressing RPL26-mediated augmentation of p53 protein synthesis [64]. Another example is RPL11 that not only stabilizes p53 but also boosts p53 transcriptional activity [65]. It has been found that RPL11 can be recruited on the p53 target gene promoters via its binding to MDM2, and therefore relieve MDM2-mediated transcriptional repression of p53 [65]. Interestingly, several RPs, such as RPL6, RPL26, RPS25, RPS27 and RPS27A, are found to be transcriptionally repressed by p53 or post-transcriptionally downregulated by MDM2, thereby forming a negative feedback loop [52,59,61,62,64]. A recent *in vivo* study has gracefully validated the physiological significance of RPs in ribosomal stress-triggered p53 activation. In this study, Macias *et al.* [66,67] generated mice carrying a mutation of Mdm2^C305F^ which was defective in binding to RPL5 and RPL11, and found that p53 activation in response to ribosomal stress was markedly impaired, whereas p53 response to DNA damage was normal, in these mutant mice [66].

Growing evidence has demonstrated the central role of the RP-MDM2-p53 pathway in controlling cancer development. For instance, Sasaki *et al.* [68] found that the oncogenic protein PICT1 attenuates p53 activation upon stress by sequestering RPL11 in the nucleolus and thus blocking the RPL11-MDM2 interaction. Reversely, in PICT1-deficient cells, p53 ubiquitination and degradation was dramatically repressed by increased binding of RPL11 to MDM2 [68]. Another oncoprotein SRSF1 was found to stabilize p53 by abrogating its MDM2-mediated degradation via direct binding to RPL5 [69]. In addition, the ARF tumor suppressor has been shown to activate p53 through an RP-MDM2-p53 cascade [70]. ARF can directly bind to RPL11 and this interaction enhances p53 activation in response to either oncogenic or ribosomal stress. These two nucleolar proteins work together to form a complex with MDM2 and p53, and tremendously improves the stability and activity of p53. Also, high expression of ARF causes accumulation of ribosome-free RPL11, thus bolstering the RPL11-MDM2 interaction and consequent p53 activation [70]. Ling Zhi-8 (LZ-8), an immunomodulatory protein exhibiting antitumor activity, derived from medicinal mushroom *Ganoderma lucidum* [71] can induce p53-dependent G_1_ cell cycle arrest and suppress proliferation of lung cancer cells by triggering ribosomal stress and RPS7-MDM2 binding [72]. The above studies have underscored the pivotal role of RP in connecting other important proteins to the p53 pathway. Hence, it would be interesting to profile the protein interactomes of the MDM2-binding RPs, which will largely improve our understanding of the regulatory network of the RP-MDM2-p53 pathway.

Interestingly, post-translational modifications (PTM) of RPs have been found to be essential for MDM2-p53 regulation. Under normal conditions, the NEDD8-mediated RPL11 NEDDylation promotes its nucleolar localization, whereas ribosomal stress can induce de-NEDDylation of RPL11 which subsequently relocalizes to the nucleoplasm leading to p53 activation [65,73]. In contrast, NEDDylation of RPS14 positively prompts its activity to release p53 from MDM2 inhibition [74]. Additionally, several RPs can also be ubiquitinated by MDM2 leading to either impaired or prompted p53 activation. For instance, ubiquitinated RPL26 tends to be degraded resulting in impaired p53 activation [64], whereas ubiquitinated RPS7 promotes further activation of p53 to a greater extent and elicits apoptosis upon ribosomal stress [56]. Acetylation and phosphorylation are two important events that extensively influence various cellular processes during tumor growth, including MDM2-p53 signaling [40]. Although several RPs can be acetylated (e.g., RPL24 [75]) or phosphorylated (e.g., RPS6 [76]), less attention has been paid to the possible role of acetylation or phosphorylation of RPs in the regulation of the MDM2-p53 pathway. Recently, a proteomic analysis of nucleolar protein acetylation revealed that many MDM2-binding RPs, including RPL5, RPL11, RPL26, RPS3, RPS7 and RPS27A, could potentially be acetylated [77], which implies that acetylation of these RPs may be involved in the MDM2-p53 regulation. This is certainly an intriguing and important question that would be addressed in future study.

Taken together, this gradually appreciated RP-MDM2-p53 pathway is subjected to multiple regulations, including RP-associating cofactors and PTMs, and has been playing an increasingly essential role in controlling tumorigenesis.

## 3. Roles of p53 and Mutant p53 Target Genes

It has been well documented that most p53 functions are mediated by transcriptional activation of its target genes in response to various stress signals (Figure 1), although cytoplasmic p53 was also shown to directly bind to, for example, BAX to trigger mitochondrial outer membrane permeabilization (MOMP) and apoptosis [78]. There are four sets of experimental criteria for identifying a p53 target gene [12]. First, one or several p53 responsive elements (REs) are required close to or within the gene. A consensus motif, 5'-RRRCWWGYYY-3', has been identified in p53 REs where R is a purine, Y is a pyrimidine, W is either A or T (adenine or thymine), G is guanine and C is cytosine [79,80]. A canonical p53 RE contains two consensus motifs separated by a spacer of 0–21 base pairs; Second, the mRNA level of the candidate gene must be upregulated or downregulated by p53; Third, by engineering the RE from the candidate gene into a vector encoding a reporter gene (e.g., luciferase), p53 can regulate the reporter gene expression; Finally, p53 protein specifically and directly associates with the RE in the DNA through chromatin immunoprecipitation (ChIP) assay. Thus far, hundreds of p53 target genes have been identified, which are involved in a wide variety of cellular processes, including cell cycle arrest, DNA repair, apoptosis, senescence, autophagy, metabolic homeostasis, *etc.* [12,29,81]. In cells exposed to mild stress signals, p53 induces protective and pro-survival responses, such as temporary cell cycle arrest, allowing cells to repair their DNA and maintain the genome integrity. Alternatively, intensive stress signals provoke p53-mediated irreversible responses, such as apoptosis and senescence, which eliminate irreparable damaged or malignant cells.

What accounts for p53 selective activation of its target genes dictating diverse cell fates is an intriguing question. A number of studies have demonstrated factors that regulate target gene selection by p53 [82]. Several studies suggest that p53 binds to its target gene promoters with varying affinities-promoters of target genes controlling cell cycle retain high affinity to p53, whereas apoptotic target gene promoters have low affinity [83,84,85]. It is therefore proposed that a low level of p53 induced by mild stress signals is only able to activate target genes involved in cell cycle control, while a high level of p53 stimulated by potent stress signals can activate apoptotic target genes. However, another study reported that a portion of apoptotic genes, such as *PUMA*, *p53AIP1* and *NOXA*, also have high-affinity p53 REs [86]. Hence, other mechanisms must also be responsible for p53 target gene selectivity. Upon stress signals, p53 undergoes a series of PTMs which have been shown to play an essential role in p53-dependent cell fate determination. For example, Ser-46 phosphorylation is required for p53-dependent transcriptional activation of the apoptotic gene *p53AIP1*, and Ser-46 to Alanine mutation that prevents phosphorylation of this site abolishes p53-mediated activation of *p53AIP1*, but not the cell cycle gene *p21* [87]. Another example is acetylation of Lys-120 that is crucial to p53-triggered apoptosis. It has been found that Lys-120 to Arginine mutant retains the ability to transcriptionally activate *p21*, but fails to induce the expression of the apoptotic target genes, *PUMA* and *BAX* [38,39]. Additionally, several p53-interacting proteins have also been shown to control specific transactivation of p53 target genes. One of the prominent examples is the ASPP protein family, including ASPP1, ASPP2 and iASPP, that directly binds to the central core domain of p53 [88]. The association of ASPP1 and ASPP2 with p53 specifically prompts p53-dependent apoptotic gene activation, while iASPP opposes this effect [88,89]. Altogether, numerous mechanisms have been found to be associated with selective activation of target genes by p53, and thorough elaboration of these mechanisms of cell fate determination would clearly contribute to the development of therapeutic strategies for human cancer.

Aside from the aforementioned protein-encoding genes, p53 can also induce non-coding RNA genes, including microRNA (miRNA) and long non-coding RNA genes. miRNAs are small noncoding RNAs, 9–22 nucleotides in length, which usually bind to the 3' UTR of target mRNAs and post-transcriptionally silence their expression. Principally, the generation of a functional miRNA includes two steps, transcription and maturation. RNA Pol II or RNA Pol III-mediated transcription produces the miRNA precursor, pri-miRNA, which undergoes further processing to generate mature miRNA. Only the mature miRNA is employed to form the RNA-induced silencing complex (RISC) to inhibit the target gene expression [90]. Interestingly, p53 has been found to not only transcriptionally regulate miRNA gene expression [91], but also modulate the maturation of miRNA [92]. The processing of pri-miRNA requires the nuclear RNase III Drosha complex which contains Drosha, DGCR8 and several RNA-associated proteins including the DEAD box RNA helicases p68. A recent study has shown that p53 is able to facilitate the formation of the Drosha complex by directly associating with p68, consequently enhancing the maturation of several tumor suppressive miRNAs [92]. Thus far, a number of p53-regulated miRNAs have been identified and shown to be involved in multiple cellular processes, including cell cycle progression, DNA repair, cell survival, epithelial-mesenchymal transition (EMT), stemness, metabolism and angiogenesis [93,94]. For example, miR-34a–c, a family of miRNAs transcriptionally activated by p53, have been shown to elicit cell cycle arrest by targeting cell cycle-related genes, such as, CDK4, CDK6, CCNE2 and MET [95,96]. Also miR-34s can induce apoptosis by down-regulating several well-documented anti-apoptotic genes, including BCL2, BIRC3 and DcR3 [96,97,98]. Notably, the tumor suppressive function of miR-34s also attributes to its activity to inhibit other important oncogenic pathway, including MYC [99], E2F [100], Wnt [101], Snail1 [102], MAGE-A [103] and c-Kit [104]. Of interest, several studies revealed that p53 also transcriptionally induces large intergenic non-coding RNAs (LincRNAs) [105,106], which are regarded as master gene regulators involving chromatin remodeling and histone modifications. For instance, lincRNA-p21 can be activated by p53 and serves as a global repressor of oncogenic genes thus leading to the induction of apoptosis [106].

When talking about wild-type p53, it is impossible not to mention mutant p53. Most cancer-associated inactivation of p53 results from missense mutations, single base-pair substitutions leading to the translation of a different amino acid that may either annihilate the wild-type p53 function or endow the mutant p53 with new functions. A seminal study has recently found that the elevated expression of the DNA cytosine deaminase APOBEC3B in breast cancer may contribute to the mutagenesis of p53 [107]. Because of the essential role of p53 in controlling cell survival as a transcription factor, it is interesting, but not surprising, that the vast majority of the missense mutations occur in the DNA binding domain (DBD) of p53. Among these mutations, the six most frequently mutated residues, including R175, G245, R248, R249, R273 and R282, are regarded as “hot spot” residues [16,18]. Mutations in the DBD disrupt the DNA binding ability of p53, thus mutant p53 usually loses transcriptional activity and, in many cases, causes a dominant-negative effect on the remaining wild-type allele through forming mutant/wild-type p53 co-tetramers [108] or supra-tetrameric aggregates [109]. Most interestingly, studies have demonstrated that mutant p53 may also acquire “gain-of-function”, not present in wild-type p53. Principally, four mechanisms underlie gain-of-function of mutant p53 [16,17,18]. Firstly, mutant p53 can directly bind to p53 family proteins, p63 and p73, and inhibit their transcriptional activity [110,111,112,113]. Secondly, the interaction of mutant p53 with other transcription factors, such as NF-Y [114], SREBP-2 [115], Sp1 [116], ETS1 [117], ETS2 [118] and VDR [119], leads to indirect regulation of their target genes. Most of the known mutant p53 target genes are transcriptionally regulated via this indirect manner. Thirdly, mutant p53 can recognize and associate with structure-specific DNA, e.g., nuclear matrix attachment regions (MARs) [120,121], resulting in transcriptional modulation of target gene expression. Lastly, mutant p53 can also bind to non-transcription factors, such as TopBP1 [122], Pin1 [123] and others [18]. A large number of mutant p53 target genes have been identified and found to support tumor cell survival and proliferation by inhibiting apoptosis, promoting chemoresistance and regulating metabolism as well as cell-cell/cell-extracellular matrix (ECM) signaling [18]. In addition to these target gene-mediated functions, mutant p53 has also been shown to contribute to genomic instability, inflammation, migration, invasion, angiogenesis and metastasis [17,18], which places it in the core position of tumorigenesis. Therefore, targeting mutant p53 could be an important therapeutic strategy, with great promise, for human cancers bearing mutant p53.

## 4. Drug Development 

As the essential role of p53 is killing tumor cells, numerous strategies have been developed to activate p53 or restore mutant p53 function for cancer therapy. Although some traditional genotoxic chemotherapeutic drugs, such as Adriamycin and platinum-based drugs, activate the p53 pathway, they also cause systemic toxicity and induce multidrug resistance. It is therefore critically important that non-genotoxic chemotherapeutics be developed to specifically target p53. Because of the central role of MDM2 in modulating p53 activity, much attention has been devoted to developing MDM2 inhibitors that can specifically release p53 from MDM2 repression. The ground-breaking work in this field is the development of Nutlin, a small molecule disrupting MDM2-p53 interaction without causing genotoxicity [124]. It has been shown that Nutlin mimics a MDM2-binding p53 peptide that competitively associates with MDM2 and prevents MDM2-p53 interaction, resulting in robust non-genotoxic activation of p53 [124]. The Nutlin derivative RG7112 (RO5045337) has been developed by Hoffmann-La Roche and is the first specific p53 activator that advanced to clinical trials. It is a more potent inhibitor of MDM2-p53 interaction, yet through the similar mechanism, compared to Nutlin [125]. There are also other promising compounds that antagonize MDM2 function entering Phase I trials [126], including RO5503781, SAR405838, CGM097 and MK-8242 (Table 1).

One of the important negative regulators, the SIRT1 deacetylase, has been shown to maintain the deacetylated status of p53, and thus promote its MDM2-mediated degradation [127,128]. It has also been revealed that developing compounds against SIRT1 could be an alternative strategy for activation of p53. For example, Tenovins can inhibit activities of SIRT1 and SIRT2, and thereby stabilize and activate p53. Although Tenovins suppress growth of p53-null or mutant cancer cells, wild-type p53 do sensitize cells to Tenovins-induced apoptosis [129]. Another compound, Inauhzin, has recently been identified as a specific SIRT1 inhibitor. It can directly bind to SIRT1 and inhibit its deacetylase activity leading to dramatic p53 activation and p53-dependent apoptosis and cell growth arrest at a very low concentration of two micromolar. This study also showed that Inauhzin does not bind to DNA and is thus unable to cause genotoxicity [130]. Importantly, both compounds were shown to inhibit growth of xenograft tumors in mice [129,130], indicating that they are potential drug candidates that require further preclinical studies.

As mutations in p53 occur in up to 50% of human cancers, how to restore mutant p53 protein to a transcriptionally functional conformation becomes a hot topic and promising strategy for the treatment of cancers harboring mutant p53. One of the most successful compounds is PRIMA-1^MET^ (also known as APR-246). While PRIMA-1^MET^ can induce cell death independently of p53 [131,132,133], it has been found to restore mutant p53 (R273H and R175H) function *in vitro* and *in vivo* by covalently binding to mutant p53 and mediating modification of its thiol groups, and has completed a Phase I clinical trial [134,135,136,137]. Fersht and colleagues designed two compounds, PK083 and PK7088, to target mutant p53 Y220C [138,139]. They showed that these two compounds raise the melting temperature of mutant p53 and restore mutant p53 protein to a wild-type conformation, consequently leading to cell cycle arrest, apoptosis and growth inhibition [138,139]. Recently, the thiosemicarbazone family compound NSC319726 has been identified to convert p53^R175^ mutant into a wild-type structure by chelating the zinc ion and changing the redox state, and therefore rescues transcriptional function of p53^R175^ mutant resulting in tumor suppression [140].

There are certainly many other compounds, in addition to those mentioned above, targeting p53 or the mutant p53 pathway [141]; however, it requires great efforts and often takes a very long time to translate the basic biomedical research into effective medicines. The more detailed the elucidation of the molecular basis of p53 activation is, the greater the chance of more efficient and specific therapeutic strategies being developed for the benefit of patients with cancer.

**Table 1:** Summary of compounds activating p53 or reactivating mutant p53 discussed in the essay.

ID	Company	Status	Mechanism	*In vivo* Test	References
RG7112 (RO5045337)	Hoffmann-La Roche	Phase I	Inhibition of MDM2-p53 interaction	Advanced malignancies, except leukemia	[125]
MI-773 (SAR405838)	Sanofi-Aventis	Phase I	Inhibition of MDM2-p53 interaction	Advanced cancer	[126]
CGM097	Novartis	Phase I	Inhibition of MDM2-p53 interaction	Selected advanced and refractory solid tumors	[126]
MK-8242	Merck	Phase I	Inhibition of MDM2-p53 interaction	-	[126]
RO5503781	Hoffmann-La Roche	Phase I	Inhibition of MDM2-p53 interaction	Soft tissue sarcoma; leukemia	[142]
Tenovin	-	Preclinical	Inhibition of Sirt1 and Sirt2 activity	-	[129]
Inauhzin	-	Preclinical	Inhibition of Sirt1 activity	-	[130]
PRIMA-1^MET^/APR-246	Aprea AB	Phase I/II	Reactivate mutant p53	Refractory hematologic malignancies and prostate cancer	[136]
PK083	-	Preclinical	Reactivate mutant p53 Y220C	-	[138]
PK7088	-	Preclinical	Reactivate mutant p53 Y220C	-	[139]
NSC319726	-	Preclinical	Reactivate mutant p53 R175H	-	[140]

## 5. Conclusions and Challenges

As one of the most important tumor suppressors, p53 can be activated in response to multiple stress signals leading to transcriptional activation of a large group of target genes and consequently suppression of tumorigenesis principally through inducing cell cycle arrest, senescence, apoptosis and metabolic changes. Therefore, the p53 pathway has become an ideal target for the development of chemotherapeutics for cancer. While the achievements in dissecting the p53 signaling pathway opens a promising avenue which might lead to our final success in the battle against cancer, these seminal studies also raise a great many questions. For example, acetylation of several lysine residues, such as Lys-120 and Lys-164, are critical for transcriptional activity of p53. To assess whether full activity of p53 is essential for its tumor suppressive ability, an animal model with triple lysine to arginine mutations of Lys-117, Lys-161 and Lys-162 (equivalent to human Lys-120, Lys-164 and Gln-165) was generated [143]. In the study, p53^3KR^ was shown to partially lose its transcriptional activity leading to abrogated cell cycle arrest, apoptosis and senescence upon DNA damage while retaining tumor suppressive function by inducing a range of target genes involved in metabolic regulation [143]. Another study showed that inactivation of TAD1, one of the two distinct transactivation domains (TADs) of p53, abolishes acute DNA damage-triggered p53 activation whereas the tumor suppressive function of p53 remains intact [144,145]. Therefore, it is interesting and challenging whether activation of metabolic target genes is sufficient for p53-dependent tumor suppression, and whether therapeutic strategies can be developed by partially attenuating p53 activity and thus reducing DNA damage-caused, p53-dependent detrimental side effects on normal tissues. Additionally, more questions are posed based on what has been learned about p53. Do any other stress signals, besides those mentioned above, activate p53 pathway? How many RPs can bind to MDM2 upon ribosomal stress? What other target genes does p53 regulate? Does p53 regulate the chromatin landscape of its target genes? What is the mechanism underlying the frequent mutation of the “hot spot” residues of p53 gene? Is there any stress signal that can repress mutant p53 function? Do PTMs regulate mutant p53 functions? Can we design compounds that specifically control tumor cell fate? Hence, tremendous efforts are needed to elucidate these enigmas, and thereby hopefully lead to the development of effective compounds or strategies to treat cancer.
